# Cancer Incidence in World Trade Center Rescue and Recovery Workers, 2001–2008

**DOI:** 10.1289/ehp.1205894

**Published:** 2013-04-23

**Authors:** Samara Solan, Sylvan Wallenstein, Moshe Shapiro, Susan L. Teitelbaum, Lori Stevenson, Anne Kochman, Julia Kaplan, Cornelia Dellenbaugh, Amy Kahn, F. Noah Biro, Michael Crane, Laura Crowley, Janice Gabrilove, Lou Gonsalves, Denise Harrison, Robin Herbert, Benjamin Luft, Steven B. Markowitz, Jacqueline Moline, Xiaoling Niu, Henry Sacks, Gauri Shukla, Iris Udasin, Roberto G. Lucchini, Paolo Boffetta, Philip J. Landrigan

**Affiliations:** 1 Department of Preventive Medicine, Icahn School of Medicine at Mount Sinai, New York, New York, USA; 2 New York State Cancer Registry, New York State Department of Health, Albany, New York, USA; 3 Department of Medicine, Icahn School of Medicine at Mount Sinai, New York, New York, USA; 4 Connecticut Tumor Registry, State of Connecticut Department of Public Health, Hartford, Connecticut, USA; 5 Department of Medicine, Bellevue Hospital Center/New York University School of Medicine, New York, New York, USA; 6 Department of Medicine, State University of New York at Stony Brook, Stony Brook, New York, USA; 7 Queens College, City University of New York, Flushing, New York, USA; 8 Department of Population Health, Hofstra North Shore–LIJ School of Medicine, Great Neck, New York, USA; 9 New Jersey State Cancer Registry, New Jersey Department of Health, Trenton, New Jersey, USA; 10 Department of Environmental and Occupational Medicine, University of Medicine and Dentistry of New Jersey–Robert Wood Johnson Medical School, Piscataway, New Jersey, USA; 11 Department of Occupational Medicine, University of Brescia, Brescia, Lombardy, Italy; 12 Tisch Cancer Institute and Institute for Translational Epidemiology, Icahn School of Medicine at Mount Sinai, New York, New York, USA

**Keywords:** cancer, cancer incidence, cancer registry, epidemiology, September 11th, World Trade Center, WTC Health Program

## Abstract

Background: World Trade Center (WTC) rescue and recovery workers were exposed to a complex mix of pollutants and carcinogens.

Objective: The purpose of this investigation was to evaluate cancer incidence in responders during the first 7 years after 11 September 2001.

Methods: Cancers among 20,984 consented participants in the WTC Health Program were identified through linkage to state tumor registries in New York, New Jersey, Connecticut, and Pennsylvania. Standardized incidence ratios (SIRs) were calculated to compare cancers diagnosed in responders to predicted numbers for the general population. Multivariate regression models were used to estimate associations with degree of exposure.

Results: A total of 575 cancers were diagnosed in 552 individuals. Increases above registry-based expectations were noted for all cancer sites combined (SIR = 1.15; 95% CI: 1.06, 1.25), thyroid cancer (SIR = 2.39; 95% CI: 1.70, 3.27), prostate cancer (SIR = 1.21; 95% CI: 1.01, 1.44), combined hematopoietic and lymphoid cancers (SIR = 1.36; 95% CI: 1.07, 1.71), and soft tissue cancers (SIR = 2.26; 95% CI: 1.13, 4.05). When restricted to 302 cancers diagnosed ≥ 6 months after enrollment, the SIR for all cancers decreased to 1.06 (95% CI: 0.94, 1.18), but thyroid and prostate cancer diagnoses remained greater than expected. All cancers combined were increased in very highly exposed responders and among those exposed to significant amounts of dust, compared with responders who reported lower levels of exposure.

Conclusion: Estimates should be interpreted with caution given the short follow-up and long latency period for most cancers, the intensive medical surveillance of this cohort, and the small numbers of cancers at specific sites. However, our findings highlight the need for continued follow-up and surveillance of WTC responders.

Recent studies have documented the persistence of physical and mental health problems among rescue and recovery workers exposed to the World Trade Center (WTC) sites (Mauer et al. 2010; Skloot et al. 2009; Wisnivesky et al. 2011). A study of 27,449 WTC responders found persistence through 2010 of multiple physical and mental health problems including asthma, sinusitis, gastroesophageal reflux disease, depression, anxiety, and posttraumatic stress disorder (Wisnivesky et al. 2011).

Concern has arisen about the potential for increased risk of cancer among WTC responders. These men and women sustained exposures to a complex mix of toxic chemicals that included multiple known and suspected human carcinogens (Lioy et al. 2002). The combustion of jet fuel at high temperatures released soot, metals, benzene and other volatile organic compounds, and strong inorganic acids. The burning and subsequent collapse of the towers resulted in the release of particulate matter comprising asbestos; silica; cement dust; glass fibers; heavy metals including arsenic, beryllium, cadmium, chromium VI, and nickel; polycyclic aromatic hydrocarbons; polychlorinated biphenyls; and polychlorinated dibenzofurans and dibenzodioxins (Edelman et al. 2003; Lioy et al. 2002; Litten et al. 2003; McGee et al. 2003; Offenberg et al. 2003).

Four studies to date have investigated cancer in WTC responders. In 2009, the WTC Health Program published a case series of multiple myeloma cases in WTC responders, including the unusual occurrence of four cases diagnosed before the age of 45 years (Moline et al. 2009). The second study, by the Fire Department of New York City (FDNY), investigated cancer among 9,853 firefighters enrolled in the FDNY WTC Health Program (Zeig-Owens et al. 2011). This study reported an increase in the incidence of cancer in WTC-exposed firefighters compared with nonexposed firefighters, but did not present associations according to levels of WTC-related exposure. A mortality study of the New York City (NYC) Department of Health and Mental Hygiene (DOHMH) WTC Health Registry did not find an increase in standardized mortality ratios (SMRs) for any cancers or for all-cause mortality, compared with the general population (Jordan et al. 2011). Most recently, the NYC DOHMH investigated cancer incidence during 2003–2008 in a cohort of approximately 56,000 individuals registered with the WTC Health Registry and reported statistically significant increases in thyroid cancer, prostate cancer, and multiple myeloma among rescue and recovery workers in 2007–2008 (Li et al. 2012).

The purpose of the present investigation was to compare cancer incidence in a cohort of approximately 20,000 rescue and recovery workers enrolled in the WTC Health Program to the incidence in the general population during the 7 years after 11 September 2001 (9/11) and to estimate associations according to levels of WTC-related exposure.

## Methods

*Study population*. Those who participated (as employees and/or volunteers) in the rescue, recovery, and cleanup efforts at Ground Zero after 9/11 were enrolled in the World Trade Center Health Program (WTCHP) on the basis of eligibility criteria including type of duties, site location, and dates and hours worked. Details of program and eligibility criteria have been described previously (Herbert et al. 2006; Moline et al. 2008; Wisnivesky et al. 2011). The medical protocol for the monitoring program, which began in July 2002, includes self-administered physical and mental health questionnaires followed by a physical examination, laboratory tests, spirometry, and a chest radiograph. Routine WTC monitoring visits, scheduled every 12–18 months, are performed by the WTCHP clinical centers. All participating review boards including Icahn School of Medicine at Mount Sinai, New York University School of Medicine, Queens College, Stony Brook University, University of Medicine and Dentistry of New Jersey, New York State Department of Health, Connecticut Department of Health, and Pennsylvania Department of Health approved the data linkages with state cancer registries. Informed consent for research and data aggregation, for which participation was voluntary, was obtained at a monitoring visit. Data from the clinical centers were collated, prepared, and analyzed by the WTCHP Data Center.

In addition to the WTCHP, there are three clinical programs that monitor individuals for WTC-related health conditions: the FDNY (2012), the WTC Environmental Health Center (or Survivor) Program at Bellevue (WTC Environmental Health Center 2013), and the National Responders and Survivors Program (Centers for Disease Control and Prevention 2010). In addition, the NYC DOHMH has established a registry which gathers information from lower Manhattan residents, schoolchildren, building occupants, passersby, and rescue and recovery workers (WTC Health Registry 2013). The overlap in participation among the WTCHP and the FDNY, WTC Environmental Health Center, and National Responders and Survivors Programs is minimal (approximately 1% or less) due to differing enrollment criteria among these programs. Although the WTCHP and NYC DOHMH WTC Health Registry have distinct enrollment criteria and definitions for “responders,” approximately 20% of responders enrolled in the WTCHP are also registered with the NYC DOHMH WTC Health Registry.

*Case identification*. Cancer case identification was performed through linkage with the state tumor registries of New York, New Jersey, Connecticut, and Pennsylvania, each of which has attained “gold” certification by the North American Association of Central Cancer Registries for data completeness and quality. These four states account for 98% of the residences for responders at time of enrollment in the WTCHP. Information on all consented responders who enrolled in the WTCHP from its inception on 16 July 2002 until 31 December 2008 and who resided in one of these four states (*n* = 20,984) was provided to each registry, including Social Security number (SSN) where available (37%), last name, first name, sex, race/ethnicity, complete date of birth, and address at registration. At the time of the linkage, case ascertainment at all four cancer registries was considered provisionally complete through 2008.

Matching methodology varied slightly across the four state cancer registries. Each registry used a probabilistic algorithm to identify matches based on SSN (when available), date of birth, last name, first name, middle name, sex, address, and race/ethnicity, and assigned scores based on the strength of the match. NY, CT, and PA cancer registry staff performed additional manual reviews of records from other sources to resolve matches that received low scores based on the strength of the match. To assess the completeness of the linkage, we identified self-reported WTCHP cases that were diagnosed between 12 September 2001 and 31 December 2008 and subsequently confirmed by medical records, but that were not reported by any of the state tumor registries. Only cancer cases validated by one of the four state cancer registries were included in the present analyses.

Specific categories that were used for analyses were based on the groupings standardized by the National Cancer Institute’s (NCI) Surveillance, Epidemiology and End Results Program (SEER) for national cancer surveillance (NCI 2003). All identified cancer diagnoses were considered together under the category “All Cancer Sites.”

*Exposure assessment and demographic information*. Quantitative exposure measurements, especially on exposures in the first days after 9/11, are only minimally available. Data for WTC-related exposures were therefore obtained from a questionnaire administered by trained interviewers focusing on the following five variables:

Occupation. Pre-9/11 occupation was coded to the first decimal of the Standard Occupational Classification (SOC) (U.S. Office of Federal Policy and Standards 2000). SOC codes were combined to create four groups: protective services (e.g., law enforcement and emergency medical services workers); construction; buildings and grounds cleaning and maintenance and electrical, telecommunications and other installation and repair groups (CM&IRG); and all other occupations (Woskie et al. 2011). Pre-9/11 occupation is related to the tasks performed on the WTC sites; work on further classifying responder tasks is ongoing.
Exposure to dust cloud. Responders were asked if they were present south of Canal Street on 9/11 (regardless of when they started their responder duties) and, if present, whether they were engulfed in the dust cloud, exposed to significant amounts of dust but not engulfed in the cloud, exposed to some dust, or not exposed to dust.
Duration on site (days). Information was obtained from each responder on the total time spent working on site.
Work on debris pile. Responders were coded as working on the debris pile if they spent the majority of any of four time periods (September 2001, October 2001, November–December 2001, January–June 2002) working on the pile.
Exposure level. An integrated exposure variable using a 4-point scale (very high, high, intermediate, and low) was created based on total time spent working at Ground Zero, exposure to the dust cloud, and work on the debris pile (Wisnivesky et al. 2011).

Data for age as of 9/11, sex, race/ethnicity, and state of residence were collected via self-administered questionnaires at the time of enrollment or the first monitoring visit.

*Statistical analysis*. We first computed the expected incidence of each cancer outcome for each cohort member based on yearly incidence rates according to their age (in 5-year groups, e.g., 35–39 years), sex, and race/ethnicity for each year at risk (or partial year at risk) from 2001 to 2008. All state specific rates were extracted using SEER*Stat 7.0.5 (NCI 2011). New York State Cancer Registry data were used to derive expected numbers for New York residents, state-specific incidence data were used for New Jersey and Connecticut residents; and national data were used for Pennsylvania residents (NCI 2010; New York State Cancer Registry 2010). For each individual, yearly expected cancers were summed over the years at risk for that individual. Deceased participants were identified through linkage with the National Death Index (http://www.cdc.gov/nchs/ndi.htm) or next-of-kin reports to the WTCHP, and time at risk was censored at death or 31 December 2008, whichever came first. Standardized incidence ratios (SIRs) were calculated by taking the ratio of observed and expected number of cancers for each site. We performed the same analysis for all sites combined and, consistent with SEER definition for incidence (Howlader et. 2011), evaluated the number of cancers per responder in concordance with how the expected number of cases was determined. The 95% CIs for SIRs were estimated using standardized methods (Sahai and Khurshid 1993). (To the extent that the method is based on independent cancers, there could be a slight degree of approximation when it is used for multiple cancers per responder.) In addition to estimating SIRs for all cancers, we estimated SIRs after excluding cancers diagnosed within 6 months of registration into the WTCHP. SIRs were not estimated for cancer outcomes with five or fewer cases observed.

We used a multivariable generalized linear regression using Poisson models incorporating externally standardized incidence rates (Breslow and Day 1987) to adjust the SIR for all the variables listed in Table 1. Two separate regression models were employed and both utilized the first seven variables on Table 1 (sex, age on 9/11, race, smoking history, year of registration/SSN, clinical center, and occupation). (An SSN effect was extracted from the three-level SSN/year of registration variable by focusing on a specific contrast, so that this effect is based on 2001–2005 registrants.) The first model used the four-level exposure index (Wisnivesky et al. 2011) derived from the three primary exposure variables (dust exposure, duration, and worked on pile), whereas the second model directly used the three primary exposure variables.

**Table 1:** Selected characteristics of the WTCHP responders (*n* = 20,984).

Characteristic	*n* (%)
Sex
Male	17,781 (85)
Female	3,203 (15)
Median age on 9/11/2001 (years)	38
Age on 9/11/2001 (years)
< 40	11,835 (56)
≥ 40	9,149 (44)
Race/ethnicity
Black	2,698 (13)
White non-Hispanic	12,337 (59)
White Hispanic	1,345 (6)
Hispanic missing race	3,896 (19)
Other race	553 (3)
Unknown/missing	155 (1)
Smoking history
Current	3,374 (16)
Former	5,054 (24)
Never	12,240 (58)
Missing	316 (2)
Year of registration/SSN
2001–2005 with SSN	7,805 (37)
2001–2005 without SSN	5,254 (25)
2006–2008	7,925 (38)
Clinical center at time of first visit
Bellevue	1,046 (5)
Mount Sinai	15,533 (74)
Queens	1,639 (8)
Stony Brook	2,082 (10)
UMDNJ	684 (3)
Occupation
Protective services	8,765 (42)
Construction	5,138 (24)
CM&IRG	2,084 (10)
Other	4,019 (19)
Missing	978 (5)
Exposure index^*a*^
Very high	638 (3)
High	3,546 (17)
Intermediate	13,638 (65)
Low	2,728 (13)
Missing	434 (2)
Dust cloud exposure
Directly in dust cloud	4,211 (20)
Significant dust	3,415 (16)
Some dust	1,510 (7)
No dust/early arrival (9/11–9/14)	6,054 (29)
No dust/late arrival (9/15 and beyond)	5,124 (24)
Missing	670 (3)
Duration on site (days)
0–14	4,551 (22)
15–59	6,124 (29)
60–119	4,956 (24)
≥ 120	5,303 (25)
Missing	50 (0)
Location of work
On debris pile	7,403 (35)
Not on debris pile	12,865 (61)
Missing	716 (3)
CM&IRG, buildings and grounds cleaning and maintenance and electrical, telecommunications and other installa­tion and repair groups. ^***a***^Responders were in one of the two highest categories if they were directly in the dust cloud on 9/11. They were in the very high category if, in addition to being directly in the dust cloud they worked on the pile and worked on the site for ≥ 90 days. Responders were in the inter­mediate or low category if they were not directly exposed to the dust cloud on 9/11. Responders were in the low category if, in addition to not being directly exposed to the dust cloud, they also did not work on the pile and worked for < 40 days.	

Responders with missing values for any of the variables (except occupation) were excluded from multivariable models, but were included when SIRs were estimated. For occupation, since we already had a heterogeneous “other” category, we formed the reference category by combining the missing occupation with the “all other” category.

All multivariable models were repeated after excluding person-time and cancers diagnosed within 6 months of registration into the WTCHP. In general, for each variable with more than two levels, the reference category was that of anticipated lowest risk. Relative risk (RR), the ratio of adjusted SIR for a particular level relative to the adjusted SIR for a reference level, is provided for each of the exposure variable levels. Because there is no natural reference category for occupation, we performed an overall test of difference in RRs across the four levels.

We used PROC GENMOD (version 9.2; SAS Institute Inc., Cary, NC, USA) to perform Poisson models with a log link and offsets for the log of the externally standardized incidence rate for each cancer outcome. An α of 0.05 was used to determine statistical significance, and tests were two-tailed.

## Results

Responders were primarily male (85%), white non-Hispanic (59%), and never smokers (58%), and had a median age of 38 years on 9/11 (Table 1). The most common occupations were protective services and construction. Forty-three percent of responders were exposed to the dust cloud on 9/11. The median duration of service on site was 57 days.

Through linkage with the four state cancer registries we identified 575 tumor diagnoses between 9/11 and 31 December 2008 in 552 individuals from a total of 20,984 consented responders. We estimated a 15% increase in all cancer sites combined among all responders (SIR = 1.15; 95% CI: 1.06, 1.25) based on 575 cases diagnosed versus 498.8 expected (Table 2). We also estimated statistically significant increases over expected registry-based incidence rates for thyroid cancer (SIR = 2.39; 95% CI: 1.70, 3.27; 39 observed, 16.3 expected), prostate cancer (SIR = 1.21; 95% CI: 1.01, 1.44; 129 observed, 106.8 expected), combined hematopoietic and lymphoid cancers (SIR = 1.36; 95% CI: 1.07, 1.71; 74 observed, 54.5 expected), and soft tissue cancers (SIR = 2.26; 95% CI: 1.13, 4.05; 11 observed, 4.9 expected). In addition, nonsignificant positive associations were estimated for non-Hodgkin lymphoma (SIR = 1.36; 95% CI: 0.96, 1.87; 8 observed, 6.6 expected) and kidney cancer (SIR = 1.39; 95% CI: 0.95, 1.98; 31 observed, 22.2 expected). Fewer than six cases of several cancer types were observed, including mesothelioma and cancers of the pancreas; nose, nasal cavity, and middle ear; larynx; and corpus uteri.

**Table 2:** SIRs of selected cancers among WTCHP responders, 2001–2008: residents of New York, Connecticut, Pennsylvania, and New Jersey (*n* = 20,984).

Cancer	Unrestricted^*a*^				Restricted^*b*^			
	Observed	Expected	SIR	95% CI	Observed	Expected	SIR	95% CI
All sites	575	498.8	1.15	1.06–1.25	302	286.1	1.06	0.94–1.18
Oral cavity and pharynx	21	17.3	1.21	0.75–1.86	10	10.0	1.00	0.48–1.84
Digestive system	86	90.8	0.95	0.76–1.17	51	52.8	0.97	0.72–1.27
Esophagus	11	6.6	1.67	0.83–2.98	7	3.9	1.77	0.71–3.65
Stomach	11	9.1	1.20	0.60–2.16	7	5.3	1.33	0.53–2.74
Colon and rectum	44	44.4	0.99	0.72–1.33	25	25.7	0.97	0.63–1.43
Liver and intrahepatic bile duct	7	11.9	0.59	0.24–1.22	6	6.9	0.86	0.32–1.88
Lung and bronchus	43	48.4	0.89	0.64–1.20	18	29.1	0.62	0.37–0.98
Soft tissue including heart	11	4.9	2.26	1.13–4.05				
Melanoma of the skin	20	21.6	0.93	0.57–1.43	12	11.9	1.01	0.52–1.77
Breast	26	28.8	0.90	0.59–1.32	11	14.9	0.74	0.37–1.32
Prostate	129	106.8	1.21	1.01–1.44	82	66.5	1.23	0.98–1.53
Testis	16	12.2	1.31	0.75–2.13	7	5.5	1.27	0.51–2.62
Urinary bladder	29	21.2	1.37	0.92–1.96	15	12.7	1.18	0.66–1.94
Kidney and renal pelvis	31	22.2	1.39	0.95–1.98	17	12.7	1.34	0.78–2.14
Brain and other nervous system	12	9.8	1.22	0.63–2.13	7	5.2	1.34	0.54–2.77
Thyroid	39	16.3	2.39	1.70–3.27	26	8.3	3.12	2.04–4.57
Hematological	74	54.5	1.36	1.07–1.71	23	29.8	0.77	0.49–1.16
Hodgkin lymphoma	8	6.6	1.21	0.52–2.38
Non-Hodgkin lymphoma	38	28.0	1.36	0.96–1.87	13	15.3	0.85	0.45–1.45
Myeloma	9	6.4	1.41	0.64–2.67
Leukemia	19	13.5	1.41	0.85–2.19
^***a***^The interval between 9/11/01 and the earlier of 31 December 2008 and time of death.^***b***^Person-time and cases starting 6 months after registration in WTCHP.								

A second analysis restricted to cases diagnosed > 6 months after enrollment was performed, and 302 cancers were identified in 290 individuals (Table 2). In this analysis, SIRs for cancer at all sites combined decreased from 1.15 to 1.06 (95% CI: 0.94, 1.18 based on 302 diagnosed cases vs. 286.1 expected), and the SIR for hematopoietic/lymphoid cancers decreased from 1.36 to 0.77 (95% CI: 0.49, 1.16; 23 observed, 29.8 expected). The SIR for prostate cancer was essentially unchanged at 1.23 (95% CI: 0.98, 1.53; 82 observed, 66.5 expected), and the SIR for thyroid cancer increased from 2.39 to 3.12 (95% CI: 2.04, 4.57; 26 observed, 8.3 expected). The SIR for lung/bronchus cancer was significantly lower than expected in the restricted analysis, decreasing from 0.89 (95% CI: 0.64, 1.20; 43 cases observed, 48.4 expected) in the unrestricted analysis to 0.62 (95% CI: 0.37, 0.98; 18 observed, 29.1 expected). In this restricted analysis, we found fewer than six cases of soft tissue cancer, Hodgkin lymphoma, multiple myeloma, and leukemia.

In the multivariate model (Table 3), with few exceptions, results did not show statistically significant associations; however, the trends in the data are noteworthy. Relative risk of all cancers in the unrestricted model was higher in rescue workers who were Protective Services and CM&IRG workers compared with all other workers (RR = 1.22; 95% CI: 0.97, 1.54 and RR = 1.27; 95% CI: 0.96, 1.69, respectively). The results for occupation in the restricted analysis were similar with an increased risk of cancer in Protective Service and CM&IRG workers (RR = 1.34; 95% CI: 0.96, 1.88 and RR = 1.32; 95% CI: 0.90, 1.94, respectively). The relative risk of all cancers combined was elevated in the very high–exposure group compared with the low-exposure group (RR 1.19; 95% CI: 0.70, 2.01 in the unrestricted analysis and RR = 1.40; 95% CI: 0.71, 2.76 in the restricted analysis). Compared with those who arrived at the WTC sites after 14 September, the incidence of all cancers for the unrestricted analysis were increased in responders who were directly exposed to the dust cloud and in those who experienced significant amounts of dust on 9/11 (RR = 1.22; 95% CI: 0.94, 1.58 and RR = 1.32; 95% CI: 1.01, 1.73, respectively). The results for the restricted analysis were similar, with an increase in cancer incidence in responders who were directly exposed to the dust cloud (RR = 1.13; 95% CI: 0.79, 1.61) and for responders who experienced significant amounts of dust (RR = 1.23; 95% CI: 0.85, 1.76). In addition, the incidence of all cancers combined was increased in responders who worked on the pile compared with those who did not in both the unrestricted and restricted analyses (RR = 1.09; 95% CI: 0.91, 1.31 and RR = 1.21; 95% CI: 0.94, 1.56, respectively). For information on the RRs for other model covariates and specific cancer sites (prostate, thyroid and hematopoeitic and lymphoid neoplasms), see Supplemental Material, Table S1 (http://dx.doi.org/10.1289/ehp.1205894).

**Table 3:** RRs (95% CIs)^*a*^ for all cancer sites associated with WTCHP exposures.

Characteristic	Broad: time at risk^*b*^	Restricted: time at risk^*c*^
Occupation^*d*^^,^^*e*^
Construction	1.00 (0.79–1.26)	1.13 (0.83–1.54)
Protective	1.22 (0.97–1.54)	1.34 (0.96–1.88)
CM&IRG	1.27 (0.96–1.69)	1.32 (0.90–1.94)
All other	1.00 (reference)	1.00 (reference)
Exposure index^*d*^
Low	1.00 (reference)	1.00 (reference)
Medium	0.93 (0.73–1.17)	0.95 (0.67–1.33)
High	1.09 (0.82–1.45)	1.06 (0.70–1.60)
Very high	1.19 (0.70–2.01)	1.40 (0.71–2.76)
Dust exposure^*f*^
Direct	1.22 (0.94–1.58)	1.13 (0.79–1.61)
Significant	1.32 (1.01–1.73)	1.23 (0.85–1.76)
Some	0.78 (0.51–1.19)	0.49 (0.23–1.01)
None/arrival 9/11–9/14	1.03 (0.81–1.31)	0.97 (0.70–1.35)
None/arrival after 9/14	1.00 (reference)	1.00 (reference)
Duration (days on site)^*f*^
0–14	1.00 (reference)	1.00 (reference)
15–60	0.78 (0.61–0.98)	0.92 (0.65–1.29)
61–119	0.73 (0.56–0.94)	0.84 (0.59–1.21)
Over 120	0.80 (0.63–1.02)	0.97 (0.70–1.36)
Worked on pile^*f*^
Yes	1.09 (0.91–1.31)	1.21 (0.94–1.56)
No	1.00 (reference)	1.00 (reference)
CM&IRG, buildings and grounds cleaning and maintenance and electrical, telecommunications and other installa­tion and repair groups. ^***a***^RR is the ratio of adjusted SIR for a particular level relative to the adjusted SIR for a reference level.^***b***^The interval between 9/11 and the earlier of 31 December 2008 and time of death.^***c***^Person-time and cases starting 6 months after registration in WTCHP.^***d***^These RRs are based on a model that includes age, sex, race/ethnicity, clinic, smoking, the three-level variable that includes SSN and year of registration, occupation, and exposure index.^***e***^The overall test of difference in RRs across the levels of occupation was 0.1392 for the broad analysis and 0.2801 for the restricted analysis.^***f***^These relative risks are based on a model that includes age, sex, race/ethnicity, clinic, smoking, the three-level variable that includes SSN and year of registration, occupation, presence in dust cloud, duration, and location.		

## Discussion

Over the 7 years following 9/11, the incidence of several cancer types in WTC responders was greater than expected, including cancer at all sites combined as well as thyroid, prostate, and combined hematopoietic and lymphoid cancers. Results of this study should be interpreted with caution given the short follow-up, long latency period associated with most cancer types, and small numbers of observed or expected cases for several of the cancer outcomes assessed.

The findings of the present study are concordant with recent studies of NYC firefighters conducted by FDNY (Zeig-Owens et al. 2011) and a study of New York State residents enrolled in the NYC DOHMH WTC Health Registry (Li et al. 2012). In comparison to the general male population of the United States, the SIR for all cancers in WTC-exposed FDNY personnel was 1.10 (95% CI: 0.98, 1.25). Similarly, in the NYC DOHMH study, the SIR for all sites combined in 2007–2008 for rescue and recovery workers was nearly significantly elevated (SIR 1.14; 95% CI: 0.99, 1.30). Both studies found elevated SIRs for thyroid, prostate, and certain hematological cancers (non-Hodgkin lymphoma in the FDNY study; multiple myeloma in the NYC DOHMH study). In addition, both studies reported that the incidence of lung cancer was lower than expected among first responders and rescue and recovery workers.

There is strong causal evidence linking thyroid cancers with exposure to iodine-131, but there is no evidence that this radionuclide was present at Ground Zero (Siemiatycki et al. 2004). Evidence for occupational risk factors of prostate cancer is very weak, and heightened diagnosis due to increased medical surveillance is a possible explanation for greater than expected numbers of prostate cancer diagnoses. It is well recognized that in heavily screened populations, prostate and endocrine cancers are diagnosed more frequently than in populations subjected to less rigorous screening (Davies and Welch 2006; Draisma et al. 2003). In this situation, asymptomatic cancers that would otherwise not be detected are detected at a higher than normal rate (Welch and Black 2010). Additionally, although these are not routinely performed during monitoring visits, responders with respiratory health problems were referred for chest computed tomography scans. This imaging is known to increase detection of incidental thyroid nodules (Swenson et al. 2003). Similarly, although our program did not measure prostate-specific antigen (PSA) levels during monitoring visits, it is possible that when responders were referred back to their medical providers, PSA levels were tested. This possibility is supported by findings in the FDNY study, in which a statistically significant increase in prostate cancer among non-WTC–exposed firefighters was observed (Zeig-Owens et al. 2011).

The short follow-up time relative to the expected latency of cancer development, the young age of the cohort, and the potential for a healthy worker effect must be considered when interpreting our findings. This analysis covers only the first 7 years after the WTC attacks, whereas most occupational cancers become manifest only ≥ 1 decades after carcinogenic exposure. Benzene exposure, a by-product of petroleum fires, however, has been shown to have the greatest magnitude of association within the first 10 years of exposure (Richardson 2008) and was found to be elevated in air samples around the WTC sites (U.S. Environmental Protection Agency 2003). Additionally, the cohort is relatively young. Thus, despite the large sample size, we had limited power due to small numbers of cases, particularly for less-common cancer outcomes. For the analyses of broad and restricted time at risk, we have 80% power to detect statistically significant SIRs of 1.13 and 1.17, respectively, for all sites, 1.27 and 1.34 for prostate cancer, and 1.38 or 1.51 for hematological cancers. It is therefore to be expected that as follow-up time increases and the population ages, many more cases of cancer will be recognized in the WTC responder population, and power to detect differences between observed and expected numbers of cases, if they are present, will increase.

WTC responders, like many employed populations, were substantially healthier than the general population at the time when they began their service at the WTC site, and were therefore at lower risk of cancer than the general U.S. population, which includes persons who are chronically ill, hospitalized, or otherwise unemployable. Indeed, the WTC responder population was arguably even more fit than most working populations because many were in occupations that required periodic physical and mental fitness tests.

Our program is voluntary, and only enrolled and consented responders were included in this analysis. Although a comprehensive roster of all WTC responders was not kept, it is estimated that approximately 50,000 individuals would have been eligible to participate (Savitz et al. 2008). If enrollment was nondifferential with respect to exposure or outcome, it would not bias our results. However, we cannot exclude the possibility that self-selection, either into or out of our program, was associated with exposure and/or cancer, leading to biased estimates of SIRs. In the sensitivity analysis that excluded cancers diagnosed within 6 months of enrollment, the estimated SIR for cancer at all sites combined decreased from 1.15 to 1.06, and the estimated SIR for combined hematopoietic/lymphoid cancers decreased from 1.36 to 0.77. Only the incidence of thyroid and prostate cancers remained higher than expected, with a significant increase estimated for thyroid cancer.

Underreporting of certain cancers to state cancer registries is another potential source of undercounting of cancer cases in our responder population. As of 27 December 2011, we had identified 18 cancer cases that were self-reported to our program by responders, confirmed through medical record review as being diagnosed in the follow-up period, but could not be successfully linked through any of the four state tumor registries. In previous studies, the most commonly underreported cancer types have been melanomas, prostate, and hematologic malignancies, which are cancers that are often diagnosed and treated outside of the hospital setting (Craig et al. 2012; Rigel 2010). Our findings are generally consistent with this pattern. Of our 18 unmatched cases, 7 were prostate cancers and 6 were hematological malignancies.

Moline et al. (2009) reviewed all cases of multiple myeloma diagnosed between 9/11 and 10 September 2007 among responders enrolled in the WTCHP that were confirmed by medical record review, regardless of whether they were reported to state cancer registries, and identified eight cases among 28,252 responders. The authors noted that the incidence of this cancer (*n* = 4) was greater than expected (1.0 as determined by SEER rates) in responders < 45 years of age. In contrast with our analysis, the case series included all respondents, including those who did not participate in a monitoring visit, and without any restrictions on state of residence at time of enrollment.

Most responders (98%) resided in New York, New Jersey, Connecticut, or Pennsylvania at the time of their enrollment in the WTCHP and would, therefore, at that time, have been covered by one of the participating cancer registries in those four states. However, cancers among responders who moved to other states before diagnosis would not have been ascertained. To address this, we are now conducting linkages to additional state cancer registries—especially in states where workers are likely to move for retirement—for use in future investigations. In addition, we analyzed the pattern of residence for last known address and found it to be similar to address at registration. And a preliminary review of cancer registry data from North Carolina and Florida, states known to have had an influx of retirees, indicated that only two additional cases would have been ascertained for present analyses.

Failure to obtain SSNs is yet another possible cause of underascertainment. The WTCHP discontinued collecting SSN in 2006 because of privacy concerns among some responders, with the potential of making linkages with state tumor registries more challenging. RRs for all cancers combined were slightly elevated among responders who provided their SSN to the program compared with responders who did not (RR = 1.17; 95% CI: 0.92, 1.47) [see Supplemental Material, Table S1 (http://dx.doi.org/10.1289/ehp.1205894)].

Exposures in the aftermath of 9/11 were unusual in terms of their high intensity and the complex mix of known and suspected carcinogens involved. These unique features of the WTC exposures complicate comparisons with effects of occupational exposures to carcinogens that may involve lower levels of exposure to a less diverse group of carcinogens over a longer period of time. Therefore, we hypothesized that high levels of exposure at Ground Zero to mixed agents might have resulted in cancers with a relatively short latency period at unexpected sites. While the NYC DOHMH study did not find a relationship between cancer incidence and levels of WTC exposure (including the cancers that had elevated SIRs in the rescue and recovery workers) (Li et al. 2012), we found evidence of an increase in all cancer sites combined among those in the very high exposure group and in responders with direct or significant indirect exposure to the dust cloud compared with responders with lower levels of exposure. Our preliminary findings highlight the need for improved exposure assessment, including exposures before 9/11, and prolonged follow-up of WTC responders to assess risks of cancer and other chronic diseases in this uniquely exposed population.

## Correction

Supplemental Table S1 did not appear in the manuscript originally published online. It has been added here.

## Supplemental Material

(549 KB) PDF
