# Supplementary material for: Cancer Incidence in World Trade Center Rescue and Recovery Workers, 2001–2008
Source: Environ Health Perspect. 2013 Apr 23;121(6):699–704. doi: 10.1289/ehp.1205894 (PMC3672914; doi:10.1289/ehp.1205894)
Supplement: (549 KB) PDF [file ehp.1205894.s001.pdf]

Environmental Health Perspectives doi:10.1289/ehp.1205894

SUPPLEMENTAL MATERIAL

Cancer Incidence in World Trade Center Rescue and Recovery Workers, 2001-2008

Samara Solan, Sylvan Wallenstein, Moshe Shapiro, Susan L. Teitelbaum, Lori Stevenson, Anne Kochman, Julia Kaplan, Cornelia Dellenbaugh, Amy Kahn, F. Noah Biro, Michael Crane, Laura Crowley, Janice Gabrilove, Lou Gonsalves, Denise Harrison, Robin Herbert, Benjamin Luft, Steven B. Markowitz, Jacqueline Moline, Xiaoling Niu, Henry Sacks, Gauri Shukla, Iris Udasin, Roberto G. Lucchini, Paolo Boffetta, and Philip J. Landrigan

Corresponding author:

Samara Solan
World Trade Center Health Program Data Center
Icahn School of Medicine at Mount Sinai Medical Center
One Gustave L. Levy Place
Box 1057
New York, NY 10029
T. 212-241-2711
F. 212-241-7235
samara.solan@mssm.edu

Supplemental Material, Table S1: RRs[a] (95% CIs) for demographics and WTCHP characteristics, for all cancer sites and site specific cancers associated with increased SIRs

Characteristic	All Cancer Sites[b]				Thyroid Cancer				Prostate Cancer				Hematopoietic/Lymphoid Cancer			
	Broad Time at Risk[c]		Restricted Time at Risk[d]		Broad Time at Risk[c,e]		Restricted Time at Risk[d,e]		Broad Time at Risk[c]		Restricted Time at Risk[d]		Broad Time at Risk[c,e]		Restricted Time at Risk[d,e]	
	RR	95% CI	RR	95% CI	RR	95% CI	RR	95% CI	RR	95% CI	RR	95% CI	RR	95% CI	RR	95% CI
**Sex[f]**																
Male	1.17	0.91-1.52	1.00	0.70-1.43	1.34	0.66-2.75	1.04	0.44-2.45					3.27	1.02-10.44	1.23	0.28-5.43
Female	1.00	reference	1.00	reference	1.00	reference	1.00	reference	NA		NA		1.00	reference	1.00	reference
**Age on 9/11[f,g]**																
<40 years	1.20	0.97-1.48	0.97	0.71-1.32	1.18	0.60-2.29	0.71	0.32-1.61	1.24	0.84-1.83	0.88	0.53-1.47	1.33	0.80-2.23	2.04	0.82-5.06
≥40 years	1.00	reference	1.00	reference	1.00	reference	1.00	reference	1.00	reference	1.00	reference	1.00	reference	1.00	reference
**Occupation[f,h]**																
Construction	1.00	0.79-1.26	1.13	0.83-1.54	1.50	0.51-4.46	2.09	0.51-8.59	1.08	0.67-1.76	0.97	0.54-1.74	0.60	0.32-1.15	0.87	0.30-2.52
Protective	1.22	0.97-1.54	1.34	0.96-1.88	2.35	0.91-6.08	4.12	1.14-14.90	1.85	1.12-3.07	1.78	0.94-3.39	0.43	0.23-0.80	0.25	0.06-1.05
CM&IRG[i]	1.27	0.96-1.69	1.32	0.90-1.94	2.18	0.66-7.15	2.35	0.47-11.66	1.87	1.09-3.22	1.65	0.84-3.23	0.86	0.40-1.83	1.31	0.38-4.49
All other	1.00	reference	1.00	reference	1.00	reference	1.00	reference	1.00	reference	1.00	reference	1.00	reference	1.00	reference
**Smoking[f]**																
Current smoker	1.14	0.88-1.46	1.43	1.04-1.96	1.82	0.82-4.08	1.94	0.76-4.94	0.63	0.32-1.25	0.63	0.28-1.42	0.38	0.15-0.97	0.41	0.09-1.81
Former smoker	1.14	0.94-1.38	1.15	0.88-1.50	1.28	0.60-2.73	0.97	0.36-2.58	1.12	0.77-1.63	0.98	0.61-1.57	0.74	0.43-1.26	0.60	0.21-1.72
Never smoker	1.00	reference	1.00	reference	1.00	reference	1.00	reference	1.00	reference	1.00	reference	1.00	reference	1.00	reference
**Social Security Number (SSN)[f]**																
SSN obtained	1.17	0.92-1.47	1.14	0.86-1.50	1.04	0.47-2.29	1.01	0.42-2.44	1.05	0.67-1.66	1.17	0.69-1.98	0.89	0.44-1.80	0.65	0.25-1.69
No SSN obtained (pre-2006)	1.00	reference	1.00	reference	1.00	reference	1.00	reference	1.00	reference	1.00	reference	1.00	reference	1.00	reference
**Year of Registration[f]**																
After 2006	1.43	1.13-1.81	1.18	0.81-1.73	0.80	0.35-1.86	1.29	0.42-4.00	1.08	0.66-1.75	1.55	0.78-3.06	2.20	1.16-4.15	1.31	0.39-4.41
2001-2005	1.00	reference	1.00	reference	1.00	reference	1.00	reference	1.00	reference	1.00	reference	1.00	reference	1.00	reference
**Exposure Index[f]**																
Low	1.00	reference	1.00	reference	1.00	reference	1.00	reference	1.00	reference	1.00	reference	1.00	reference	1.00	reference
Medium	0.93	0.73-1.17	0.95	0.67-1.33	0.54	0.24-1.23	1.01	0.29-3.46	0.69	0.43-1.09	0.65	0.36-1.15	0.86	0.45-1.64	0.95	0.27-3.34
High	1.09	0.82-1.45	1.06	0.70-1.60	0.72	0.26-1.94	1.02	0.24-4.37	1.06	0.62-1.81	1.03	0.53-1.99	1.17	0.55-2.52	0.93	0.19-4.68
Very High	1.19	0.70-2.01	1.40	0.71-2.76	0.47	0.06-3.85	1.03	0.10-10.22	0.27	0.04-2.01	0.00		1.16	0.26-5.28	2.36	0.23-23.8
**Dust Exposure[j]**																
Direct	1.22	0.94-1.58	1.13	0.79-1.61	1.02	0.39-2.67	0.66	0.21-2.10	1.54	0.89-2.66	1.53	0.78-3.01	1.34	0.65-2.76	0.98	0.26-3.71
Significant	1.32	1.01-1.73	1.23	0.85-1.76	0.82	0.28-2.38	0.54	0.15-1.94	1.75	1.00-3.08	1.98	1.00-3.91	1.17	0.53-2.57	1.13	0.30-4.24
Some	0.78	0.51-1.19	0.49	0.23-1.01	0.96	0.26-3.60	0.00		0.64	0.22-1.86	0.57	0.13-2.48	0.80	0.26-2.45	0.00	
None / arrival 9/11-9/14	1.03	0.81-1.31	0.97	0.70-1.35	0.82	0.33-2.03	0.66	0.23-1.91	1.14	0.67-1.92	1.07	0.55-2.10	0.88	0.44-1.76	0.83	0.26-2.69
None / arrival after 9/14	1.00	reference	1.00	reference	1.00	reference	1.00	reference	1.00	reference	1.00	reference	1.00	reference	1.00	reference
**Duration[j]**																
0-14 days on site	1.00	reference	1.00	reference	1.00	reference	1.00	reference	1.00	reference	1.00	reference	1.00	reference	1.00	reference
15-60 days on site	0.78	0.61-0.98	0.92	0.65-1.29	0.75	0.32-1.76	0.84	0.29-2.48	1.07	0.68-1.70	1.28	0.70-2.35	0.73	0.40-1.36	1.31	0.40-4.27
61-119 days on site	0.73	0.56-0.94	0.84	0.59-1.21	0.54	0.20-1.45	0.62	0.19-2.10	0.58	0.32-1.06	0.72	0.34-1.53	0.68	0.34-1.35	0.84	0.22-3.29
Over 120 days on site	0.80	0.63-1.02	0.97	0.70-1.36	0.65	0.26-1.65	0.71	0.22-2.28	0.71	0.42-1.19	0.85	0.44-1.64	0.48	0.23-1.00	0.68	0.17-2.64
**Worked on Pile[j]**																
Yes	1.09	0.91-1.31	1.21	0.94-1.56	0.68	0.33-1.43	1.20	0.51-2.83	0.79	0.53-1.19	0.72	0.43-1.21	1.45	0.88-2.40	2.02	0.79-5.15
No	1.00	reference	1.00	reference	1.00	reference	1.00	reference	1.00	reference	1.00	reference	1.00	reference	1.00	reference

a. RR is the ratio of adjusted SIR for a particular level relative to the adjusted SIR for a reference level.
b. In addition to the variables below, this model included race/ethnicity and clinic.
c. The interval between 9/11/01 and the earlier of 12/31/2008 and time of death.
d. Person-time and cases starting 6 months after registration in WTCHP.
e. RR should be interpreted with caution due to relatively small numbers of these cancers.
f. For prostate, thyroid, and hematopoietic/lymphoid neoplasms, these relative risks are based on a model that includes age, sex, smoking, the three-level variable that includes SSN and year of registration, occupation, and exposure index.
g. Age categories for prostate cancer only are <50 and ≥50.
h. Except for prostate cancer/broad time (p=0.026), and for hematopoietic/lymphoid cancer/broad time (p=0.046), all other p-values for overall differences between occupations exceeded 0.10.
i. Building and Grounds Cleaning and Maintenance; and Electrical, Telecommunications and Other Installation and Repair Groups.
j. For prostate, thyroid and hematopoietic/lymphoid neoplasms, these relative risks are based on a model that includes age, sex, smoking, the three-level variable that includes SSN and year of registration, occupation, dust exposure, duration and worked on pile.
